# Frequency of Home Numeracy Activities Is Differentially Related to Basic Number Processing and Calculation Skills in Kindergartners

**DOI:** 10.3389/fpsyg.2018.00340

**Published:** 2018-03-22

**Authors:** Belde Mutaf Yıldız, Delphine Sasanguie, Bert De Smedt, Bert Reynvoet

**Affiliations:** ^1^Brain and Cognition, Faculty of Psychology and Educational Sciences, KU Leuven, Leuven, Belgium; ^2^Faculty of Psychology and Educational Sciences, KU Leuven Kulak, Kortrijk, Belgium; ^3^Parenting and Special Education, Faculty of Psychology and Educational Sciences, KU Leuven, Leuven, Belgium

**Keywords:** home numeracy activities, basic number processing, calculation, kindergarteners, math achievement

## Abstract

Home numeracy has been shown to play an important role in children’s mathematical performance. However, findings are inconsistent as to which home numeracy activities are related to which mathematical skills. The present study disentangled between various mathematical abilities that were previously masked by the use of composite scores of mathematical achievement. Our aim was to shed light on the specific associations between home numeracy and various mathematical abilities. The relationships between kindergartners’ home numeracy activities, their basic number processing and calculation skills were investigated. Participants were 128 kindergartners (*M*_age_ = 5.43 years, *SD* = 0.29, range: 4.88–6.02 years) and their parents. The children completed non-symbolic and symbolic comparison tasks, non-symbolic and symbolic number line estimation tasks, mapping tasks (enumeration and connecting), and two calculation tasks. Their parents completed a home numeracy questionnaire. Results indicated small but significant associations between formal home numeracy activities that involved *more explicit teaching efforts* (i.e., identifying numerals, counting) and children’s enumeration skills. There was no correlation between formal home numeracy activities and non-symbolic number processing. Informal home numeracy activities that involved *more implicit teaching attempts*, such as “playing games” and “using numbers in daily life,” were (weakly) correlated with calculation and symbolic number line estimation, respectively. The present findings suggest that disentangling between various basic number processing and calculation skills in children might unravel specific relations with both formal and informal home numeracy activities. This might explain earlier reported contradictory findings on the association between home numeracy and mathematical abilities.

## Introduction

Cross-sectional and longitudinal research has demonstrated that individual differences in basic number processing skills are already observed before the start of primary education, and that they are related to/predictive for children’s mathematics achievement (De Smedt et al., 2009; Sasanguie et al., 2012b; Bonny and Lourenco, 2013; Libertus et al., 2013). Some of the factors that have been related to kindergarteners’ basic number processing skills are environmental. For example, the “home learning environment” refers to the opportunities provided by parents to improve their children’s overall academic success (Niklas and Schneider, 2017). More specifically, the frequency of parent-reported numeracy activities at home (e.g., counting objects, writing numbers) (LeFevre et al., 2009; Kleemans et al., 2012) or the amount of number talk observed during parent–child interactions (Levine et al., 2010), both referred to as ‘home numeracy’ (Blevins-Knabe and Austin, 2016) are associated with children’s mathematical abilities. For example, children of parents who were guided to be involved in mathematical activities at home improved in standardized mathematics achievement tests (Sheldon and Epstein, 2005). It is, however, unclear which home numeracy activities are related to which specific types of basic number processing or calculation abilities, because most of the existing studies have used composite scores, such as TEMA-2 and -3 (e.g., Blevins-Knabe and Musun-Miller, 1996; Manolitsis et al., 2013), the KeyMath test (e.g., LeFevre et al., 2009, 2010), or the Utrecht Early Numeracy Test-Revised (e.g., Kleemans et al., 2012), to measure mathematical skills. The use of these composite scores, however, might mask specific associations between home numeracy and basic number processing or calculation abilities. The aim of the current study was therefore to disentangle between subcomponents of basic number processing skills as well as calculation skills and to investigate the associations between these mathematical abilities and home numeracy. In the remainder of the introduction, we will first review studies that have focused on home numeracy and its relation to children’s mathematical skills and provide a possible explanation for the inconsistent findings. In the second section, we will discuss studies that have investigated basic number processing skills and their relation to mathematics achievement. Finally, we will describe how we investigated the relation between home numeracy, kindergarteners’ basic number processing and their calculation skills.

### Home Numeracy and Its Relation to Mathematical Skills in Children

In previous studies on the association between home numeracy and children’s mathematical skills, home numeracy has often been examined by parents’ self-reports about the frequency of numeracy activities with their children (e.g., Blevins-Knabe and Musun-Miller, 1996; LeFevre et al., 2009; but see for instance Levine et al., 2010 or Gunderson and Levine, 2011 for the use of observational measures). For example, Blevins-Knabe and Musun-Miller (1996) showed that children’s mathematical skills were *positively* correlated with some of the activities that parents reported (e.g., saying 1, 2, or 3 or mentioning number facts such as 1 + 1 = 2). On the other hand, *negative* correlations were found with other activities (e.g., using the concept ‘same number,’ showing the child how to count, and reciting the numbers 1–10). Consequently, when the frequencies of all activities were averaged, *no* significant relation was reported between home numeracy and children’s mathematical skills (Blevins-Knabe and Musun-Miller, 1996; Blevins-Knabe et al., 2000).

LeFevre et al. (2009) argued that these previous studies only focused on direct teaching efforts and neglected other indirect types of home numeracy activities, such as playing games with dice. Similar to research on home literacy (e.g., Sénéchal and LeFevre, 2002), LeFevre et al. (2009) suggested that distinguishing between *formal and informal home numeracy activities* (also referred to as ‘direct’ and ‘indirect’ activities) would improve the understanding of the relation between home numeracy and children’s mathematical skills. According to LeFevre et al. (2009), “*Direct activities* are focused on numbers and are typically used by parents for the explicit purpose of developing quantitative skills (e.g., counting objects, practicing number names, printing numbers). In contrast, *indirect activities* are real-world tasks (e.g., playing card or board games that involve numbers, cooking, or carpentry) for which the acquisition of numeracy is likely to be incidental. The crucial distinction is that, although instruction in numeracy skills also occurs during indirect activities, this instruction is embedded in a real-world task” (p. 56). With this view, LeFevre et al. (2009) argued that previous inconsistent findings on the association between home numeracy and math skills might be explained by the lack of questions that indexed informal home numeracy activities. Therefore, these authors included both formal and informal home numeracy activities in their home numeracy questionnaire. By conducting a Principal Components Analysis (PCA), they found that these activities could be categorized into four components: Two belonging to the description of ‘formal activities’ (i.e., number skills such as counting objects, and number books such as reading number storybooks) and two belonging to ‘informal activities’ (i.e., games such as playing cards games, and applications such as playing with a calculator). In this study, they also assessed children’s mathematical knowledge and mathematical fluency. The mathematical knowledge assessment consisted of a composite score of three subtests of the KeyMath test (Connolly, 2000): The numeration subtest assessed “math concepts and number system knowledge” (i.e., quantity, digit recognition, place value, and order). The addition and the subtraction subtests both started with pictures and progressed into symbolic arithmetic. Mathematical fluency was assessed by measuring the children’s latencies on single-digit addition problems. Results showed that mathematical knowledge was predicted by informal activities (i.e., games), but not by the formal ones. In contrast, mathematical fluency was predicted by both formal (i.e., number skills) and informal (i.e., games, applications) activities. LeFevre et al. (2009) concluded that experiencing informal activities at home is as important as experiencing formal activities for children in order to acquire math skills. Similarly, Niklas and Schneider (2014) showed that playing games, such as Ludo with dice (i.e., informal home numeracy) predicted kindergartners’ composite mathematics score. Furthermore, this informal home numeracy activity predicted the children’s curriculum based standardized test scores comprising nine subtests of DEMAT (“Deutscher Mathematiktest für erste Klassen” Krajewski et al., 2002) 1 year later. These effects were present even after controlling for other variables such as SES, intelligence, or rapid naming (Niklas and Schneider, 2014).

Another study, however, reported opposite findings: Formal home numeracy activities, but not the informal activities, were related to kindergarteners’ composite score on the number system knowledge subtest (LeFevre et al. (2010). Kleemans et al. (2012) also found a positive association between formal home numeracy and children’s early numeracy skills as assessed with the Utrecht Early Numeracy Test-Revised, a test measuring different numerical skills, such as comparison, estimation, counting, linking quantities, correspondence, arranging, counting quantities, sequential counting and applying knowledge of the number system (Van Luit and Van de Rijt, 2009). By contrast, Manolitsis et al. (2013) did not observe a relation between the frequency of the formal home numeracy activities and kindergartners’ knowledge of the basic math concepts, which was calculated as a composite score of four tasks of the TEMA-3 (Ginsburg and Baroody, 2003): Cardinality rule, seriation of numbers, naming of single digit numbers, and number comparison. However, Manolitsis et al. (2013) showed that the formal home numeracy activities at the beginning of kindergarten were related to counting skills. Furthermore, formal home numeracy predicted children’s math fluency at the end of first grade and this association was mediated through verbal counting abilities at the start of kindergarten.

In sum, the dissociation between formal and informal home numeracy activities alone was not enough to solve the contradictions on the relation between home numeracy and children’s mathematical skills. To further clarify this relationship, Skwarchuk et al. (2014) suggested that children’s mathematical skills might be differentially related to the types of home numeracy activities. To examine this, Skwarchuk et al. (2014) administered two measures of numerical skills: A number system knowledge test and a non-symbolic arithmetic test in which kindergarteners performed addition and subtraction trials by moving toy animals in and out a toy barn. It was hypothesized that children would use little or no knowledge of the symbolic number system during informal home numeracy activities, such as number games. Therefore, these activities were expected to be related to non-symbolic arithmetic, but not to knowledge of the symbolic number system. On the other hand, children practice numerical skills during formal home numeracy activities. Therefore, these activities were expected to be related to children’s knowledge of the number system. Indeed, the authors observed that children’s ability to represent and manipulate quantities (non-symbolic arithmetic) was uniquely predicted by informal home numeracy, whereas children’s knowledge of the number system was uniquely predicted by formal home numeracy (Skwarchuk et al., 2014). It should be noted that, in this work, informal home numeracy was operationalized as parent’s knowledge of commercially available number games for children, which makes it difficult to compare the results with previous studies on the association between parent’s reports about informal home numeracy activities and children’s numerical skills. Furthermore, children’s knowledge of the number system was indexed via a composite score of the Numeration subtest of the KeyMath test, which still did not allow a fine-grained characterization of various basic number processing skills and calculation skills.

### Basic Number Processing Skills and Their Relation to Math Achievement

It is clear that home numeracy studies have used composite math scores to index children’s mathematical skills. However, cognitive developmental studies on the building blocks of mathematical skills in children have systematically addressed the question of which basic number processing skills are the best predictors for more advanced mathematical skills (for a review, see Siegler, 2016; for a meta-analysis Schneider et al., 2017). For example, the differential role of symbolic and non-symbolic basic number processing skills as precursors of mathematical achievement has been intensively investigated (for a review, see De Smedt et al., 2013; Gebuis and Reynvoet, 2015). Non-symbolic number processing refers to the ability to comprehend, approximate, and manipulate the numerical quantity of a given set (Dehaene, 2001). Non-symbolic number processing skills have been measured with – amongst others – non-symbolic comparison (i.e., indicate the larger of two dot arrays) and non-symbolic number line estimation tasks (i.e., place a number of dots on an empty line going from, e.g., 0 to 10 dots). Cross-sectional and longitudinal studies have shown associations between children’s non-symbolic skills and mathematics achievement, addressed with both comparison (Halberda et al., 2008; Inglis et al., 2011; Libertus et al., 2011, 2013) and number line estimation tasks (Sasanguie et al., 2012b). However, the findings are not robust because there are also many studies, which have shown non-significant associations between non-symbolic number processing and math skills (for a review, see De Smedt et al., 2013).

Symbolic number processing refers to the ability to represent and use numerical symbols, such as digits or number words (Dehaene, 2011). Symbolic skills are typically measured with symbolic versions of the comparison and number line estimation tasks, in which dot arrays are replaced by digits. Correlations and predictive associations have been found between children’s mathematics achievement and symbolic number processing skills, measured with both comparison (Durand et al., 2005; De Smedt et al., 2009; Bugden and Ansari, 2011; Sasanguie et al., 2012a,b, 2013; Lyons et al., 2014; Linsen et al., 2015; Vanbinst et al., 2015a) and number line estimation tasks (Siegler and Booth, 2004; Booth and Siegler, 2006, 2008; Sasanguie et al., 2012a, 2013). Importantly, a recent meta-analysis showed that the association between symbolic comparison and math achievement was significantly larger than the association between non-symbolic comparison and math achievement (Schneider et al., 2017).

In addition to non-symbolic and symbolic number skills, mapping skills which are necessary to connect symbolic numbers with their corresponding non-symbolic representations also have been shown to be related to children’s mathematics achievement (Mundy and Gilmore, 2009; Defever et al., 2013; Brankaer et al., 2014). Mapping skills have been investigated with tasks in which children are presented with a number in one format (symbolic or non-symbolic) and are asked to indicate the equivalent number in the other format (symbolic or non-symbolic). For instance, Brankaer et al. (2014) used a mapping task in which children had to choose which of two numbers (dot arrays or digits) matched a target number (digits or dot arrays). They found that the performance in the mapping task explained part of the variance in a standardized paper-and-pencil mathematics test and in a curriculum-based mathematics test over and above the symbolic and non-symbolic comparison skills in first and third graders.

Studies that investigate whether home numeracy activities are differentially related to the above-reviewed symbolic, non-symbolic, and mapping skills are lacking. Such research is, however, necessary, because the more fine-grained assessment of children’s basic number processing and calculation skills may shed light on ambiguous associations between home numeracy and children’s mathematical skills. To our knowledge, only one study (Benavides-Varela et al., 2016) investigated the association between home numeracy and various basic number processing skills, such as exact (i.e., counting, one-to-one correspondence, and everyday numerical problems) versus approximate number processing (i.e., non-symbolic comparison and symbolic number line estimation) before. These authors observed that home numeracy was associated with children’s exact number skills but not with their approximate number processing. However, there are two differences between the study of Benavides-Varela et al. (2016) and the current one. First, in that study, home numeracy was assessed in a somewhat different way, i.e., by collecting children’s self-reports about their knowledge of number related information such as phone numbers, birth dates, and number of siblings. This operationalization makes it difficult to compare the results with those of studies making use of home numeracy questionnaires. It is unclear whether ‘home numeracy’ measured with retrieval of such numerical information from memory is an indicator of the same construct of home numeracy as reflected by a home numeracy questionnaire. Second, basic number processing skills were also assessed in slightly different ways (i.e., exact and approximate) than the symbolic, non-symbolic and mapping skills as reviewed above.

### The Current Study

In the present study, children’s non-symbolic, symbolic and mapping skills were measured with specific tasks that tapped into these different numerical abilities. Home numeracy was measured with a commonly used questionnaire (LeFevre et al., 2009) assessing both formal and informal home numeracy activities. In view of the inconsistent findings on the contributions of formal and informal home numeracy activities, we did not have *a priori* predictions regarding their differential relation with basic number processing. However, a relationship was expected between home numeracy (either formal or informal) and the children’s symbolic and mapping skills, because it is more likely that education and home numeracy correlates with symbolic compared to non-symbolic skills. In contrast, we hypothesized that the relation between home numeracy and the children’s non-symbolic number skills would be weak or absent. In line with the literature, we further hypothesized that home numeracy would be related to children’s calculation, and that symbolic number processing and mapping skills would be related to calculation skills. If the abovementioned hypotheses were confirmed, we further investigated whether the relation between home numeracy and calculation skills was mediated by symbolic number processing and mapping skills.

Children from the last (i.e., third) year of kindergarten (age range: 4.88–6.02 years) performed non-symbolic and symbolic comparison and number line estimation tasks. Their mapping skills were measured with an enumeration and a connecting task. Their calculation skills were evaluated with two calculation subtests of the TediMath (Grégoire et al., 2004). In Flanders, formal education only starts at the age of six, but nearly all of the children already enroll in a free kindergarten program, which starts when children are 2.5 years old. This program focuses on non-mandatory learning goals, such as comparing quantities, counting, ordering, and solving arithmetic operations up to number five. As a result, the tasks that were administered in the current study were age-appropriate. All children who participated in this study attended kindergarten on a permanent basis. The parents of the kindergarteners were asked to fill in the questionnaire of LeFevre et al. (2009) to assess the frequency of the numeracy activities that these children experience with their parents at home.

## Materials and Methods

### Participants

Five kindergarten schools in Flanders (Belgium) comprising ten classrooms were contacted to recruit parents and their children. In total, 160 consent forms were sent to the parents and 151 forms were returned. If the parents agreed to participate, they received the questionnaires to fill in and their children were examined at their respective schools. The return rate of the home numeracy questionnaires was 85%. Children whose parents did not return the questionnaires were excluded from further analyses (*n* = 23). An independent samples *t*-test showed that the mathematical skills of children whose parents completed the questionnaire and those who did not, did not significantly differ (*p*s > 0.36). The final sample consisted of 128 children (*M*_age_ = 5.43 years, *SD* = 0.29, range: 4.88–6.02 years; 70 females). All children had Dutch as their native language, except for five children, who had Dutch as their second language. Their knowledge of Dutch was sufficient to attend classes and to understand the task instructions. Seventy-one percent of the questionnaires were filled in by the mothers, 8% by the fathers, and in 21% of the cases the information on the informant was missing. The Socio-Economic Status (SES) of the children, as indicated by the highest educational degree of the mother, ranged from middle to high: Thirty-one percent reported to have a degree of secondary education, 34% had a bachelor or an undergraduate degree, and 30% had a master degree. For 5% of the participants, this information was missing.

### Procedure

All tasks were presented in a fixed order, on a tablet (iPad 2 Wi-Fi 16 GB with 9.7 inches display). All children first completed the enumeration and the connecting task, which investigated their mapping skills. Then, symbolic and non-symbolic number processing skills were investigated with two comparison and two number line estimation tasks. The children also completed two tasks that measured their calculation skills. Children were tested by the experimenter in a separate room at school in small groups of about four children each with their own tablet.

### Materials

#### Home Numeracy

Parents completed a Dutch translation of the questionnaire from LeFevre et al. (2009). This questionnaire consisted of questions about the frequency of engagement in various activities at home, including items on 7 general activities, 10 fine-motor activities, 3 literacy activities, and 20 numeracy activities. Parents indicated the frequency of their engagement in these activities over the past month on a 5-point scale (1 = never to 5 = everyday). The questionnaire also included demographic questions and items regarding parents’ academic expectations of their children, and their own attitudes toward mathematics and literacy. As the focus of the current study was on home numeracy, we only analyzed those items questioning the numeracy activities (*n* = 20). Additionally, two SES questions were included. In a first question, parents were asked to indicate their educational level and in a second question their monthly household income. Eighteen percent of the families did not report the monthly household income. Therefore, this question was not taken into account for further analyses. SES was solely based on maternal education level, a decision supported by the finding that the level of parental education shows a stronger association with children’s school achievement than income (Davis-Kean, 2005; Dubow et al., 2009).

#### Basic Number Processing Skills

**FIGURE 1:**
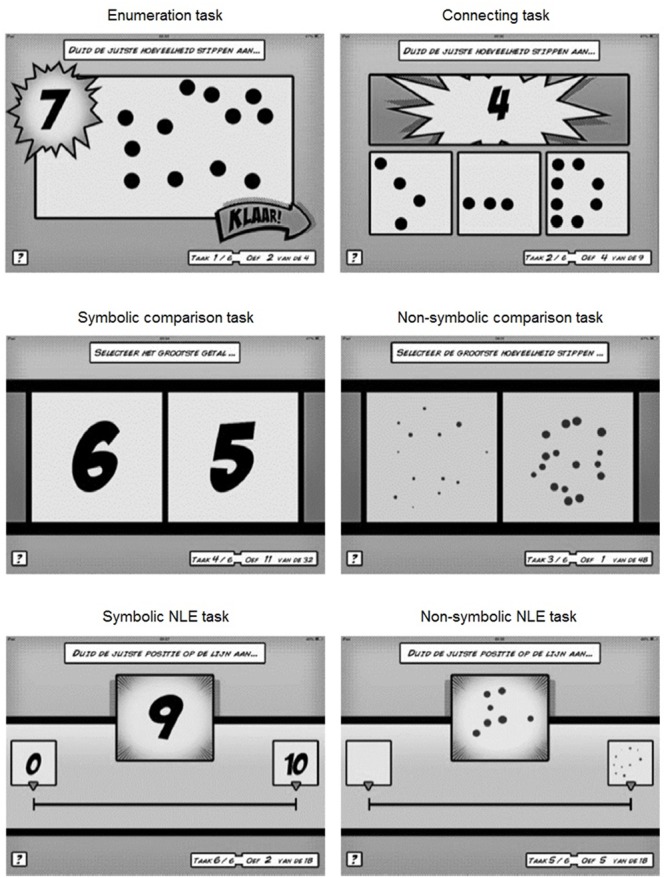
Screen shots of the mapping, comparison, and number line estimation tasks (from Maertens et al., 2016).

*Non-symbolic number processing skills* were examined using non-symbolic comparison and number line estimation tasks presented on a tablet (**Figure 1**). In the non-symbolic comparison task, the stimuli (i.e., dot arrays) were simultaneously displayed on the left and right side of the tablet screen. Children had to select the numerically larger one by tapping on the side of the numerically larger one. In all trials, one dot array was always equal to the reference numerosity 16, while the other dot array contained either 8, 11, 13, 19, 24, or 32 dots. Three ratios were presented (2.00, 1.50, and 1.20). Dot arrays were generated with the MATLAB script developed by Gebuis and Reynvoet (2011), and were controlled for four visual parameters (i.e., convex hull, total surface, item size, and density). Each combination was presented eight times, resulting in a total of 48 trials. A trial was presented for 1,500 ms, followed by a blank screen. The children had to respond during the stimulus display or during the blank screen. After the response, an inter-trial interval of 600 ms followed after which the next trial was presented. Three practice trials with feedback were presented to become familiar with the task demands. After these practice trials, no further feedback was given. The children were instructed to answer as accurately and as quickly as possible. Proportion correct was calculated as the outcome index.

In the non-symbolic number line estimation task, children had to place a number (i.e., dot array) on an empty number line by tapping where the number should go on the number line. The line was 14 cm long and labeled by an empty circle on the left side and a circle with 10 dots inside on the right. The to-be-positioned number was presented in the middle of the screen, 2.2 cm above the number line. All numbers, from 1 to 9, were shown in a random order, and they had to be positioned twice on the number line, resulting in a total of 18 trials. Three practice trials with feedback on the correct position of the target number were included. This means that children received feedback on the accuracy of the answer and that they also were informed about how close their estimation was to the target. The children were instructed to answer as accurately as possible. In line with previous studies (e.g., Booth and Siegler, 2006; Sasanguie et al., 2012a), we computed the percent absolute error (*PAE*), as the index of number line performance. The *PAE* was calculated per child by the formula of Siegler and Booth (2004):

$$\begin{array}{c} \frac{|Estimate-EstimatedQuantity|}{Scale of\mathrm{Estimates}}\times 100 \end{array}$$

For example, when a child was asked to estimate 6 on a 0–10 number line and pointed the place corresponding to 5.4, the *PAE* would be ∣(5.4–6)/10∣×100 = 6%.

*Symbolic number processing skills* were examined with symbolic comparison and number line estimation tasks (**Figure 1**). In symbolic comparison, the task requirements and design were identical to the non-symbolic comparison task, except for the stimuli. The stimuli comprised single digits 1–9. There were 16 trials with a numerical distance of 1 and 16 trials with a numerical distance of 4, resulting in a total of 32 trials. The procedure in the symbolic number line estimation task was identical to the non-symbolic number line task, except that the stimuli were digits (1–9) and the line was labeled by “0” on the left end point and by “10” on the right.

*Mapping skills* were tested using an enumeration and a connecting task (**Figure 1**). In the enumeration task, which was a variant of the Give-a-Number task (Wynn, 1990), children were shown four digits (3, 5, 7, and 9) in a random order. For each digit, they were asked to tap the collection with same number of dots on the tablet screen. The connecting task was a variant of the mapping task used by Brankaer et al. (2014). Children were shown a digit and they were asked to choose the corresponding dot array out of three by tapping on the correct one. One of the two non-matching dot arrays differed from the target number by one and the other non-matching dot array differed by two or more. All numbers, from 1 to 9, were presented once. The proportion correct trials was calculated and entered as the outcome index for both tasks.

#### Calculation Skills

Children’s calculation skills were evaluated by two calculation subtests of the TediMath (Grégoire et al., 2004), which is a multi-componential diagnostic instrument for children aged 4–8 years. The TediMath is a valid tool that discriminates between different levels of mathematical performance. Furthermore, it is a reliable instrument that the subtests the TediMath have a Cronbach’s alpha ranging from 0.70 to 0.99 (Desoete and Grégoire, 2006). The first subtest comprised of six pictorially presented single digit (numbers ranging from 2 to 7) addition (*n* = 3) and subtraction (*n* = 3) calculation questions. The experimenter read the problem to the child (e.g., “Here you see two red balloons and three blue balloons. How many balloons are there together?”). For each correct answer, the child was given one point. The second subtest comprised 18 horizontally presented both single (*n* = 10) and double (*n* = 8) digit symbolic calculation (addition) problems (e.g., 6 + 3 = ?), with numbers ranging from 0 to 45. In line with the test instructions, only the first problem was read aloud by the experimenter. The child had to solve as many problems as possible and the testing was stopped after five consecutive errors. For each subtest, the number of correctly answered problems was used to index calculation skills.

## Results

### Descriptive Statistics

We observed a low performance (*M* = 1.45, *SD* = 1.98; empirical *max* = 12) on the symbolic calculation subtest of the TediMath test, in which 51 children (40%) had none of the trials correct. Therefore, only the pictorially presented calculations subtest was used as a measure of calculation in the subsequent analyses.

There were no outliers above or below three standard deviations from the mean accuracies on the basic number processing tasks. Therefore, none of the children were removed from the analyses. Skewness and kurtosis values were within the acceptable limits for all the basic number processing and calculation tasks (skewness < 3, kurtosis < 4) (Kline, 2011). The descriptive statistics of the children’s basic number processing and calculation skills are presented in **Table 1**^1^. The children’s performance on the NLE task was typical for their age as the PAE values in the current study were comparable with those reported in previous studies, with slightly different designs, examining kindergartners (i.e., mean PAE for symbolic NLE was 0.26, [*SD* = 0.11] and for non-symbolic NLE was 0.29, [*SD* = 0.08] in the current study, which is comparable to a mean PAE of 0.24, [*SD* = 0.9] for symbolic and 0.25, [*SD* = 0.9] for non-symbolic reported in for instance Praet and Desoete, 2014; or 0.24 for symbolic and 0.21 for non-symbolic in Sella et al., 2015; or 0.24 for symbolic in Berteletti et al., 2010; or 0.27 for symbolic in Siegler and Booth, 2004).

**Table 1:** Descriptive and distribution statistics of children’s basic number processing and calculation skills.

	*M*	*SD*	Min	Max	Skewness	Kurtosis
Enumeration *(proportion correct)*	0.51	0.36	0	1	–0.18	–1.30
Connecting *(proportion correct)*	0.71	0.23	0	1	–0.90	0.42
Sym comp. *(proportion correct)*	0.66	0.16	0.31	0.94	–0.11	–0.87
Non-sym comp. *(proportion correct)*	0.59	0.10	0.40	0.83	0.40	–0.26
Sym NLE *(PAE)*	26	11	06	54	0.45	–0.16
Non-sym NLE *(PAE)*	29	08	12	51	0.12	–0.48
Pictorial calculation *(# correct)*	3.33	1.76	0	6	–0.28	–0.91
Symbolic calculation *(# correct)*	1.45	1.98	0	12	2.19	5.84

*Sym comp., symbolic comparison; Non-sym comp., non-symbolic comparison; Sym NLE, symbolic number line estimation; Non-sym NLE, non-symbolic number line estimation; PAE, percentage absolute error.*

### Home Numeracy

Similar to LeFevre et al. (2009), we first eliminated the home numeracy items that were infrequently reported in this sample. More than 60% of the parents replied “never” on the four following items: “Playing with number fridge magnets” (79.4%), “Counting down” (60.3%), “Playing with calculator” (71.3%), and “Having your child wear a watch” (68.4%). Therefore, these items were discarded from further analyses. Internal consistency of the remaining home numeracy items (*n* = 16) was 0.82, indicating that the home numeracy questionnaire was reliable.

To verify the factor structure of the home numeracy activities in our sample, we conducted a PCA on home numeracy activities with a varimax rotation, as factors were expected to be independent (see also LeFevre et al., 2009). PCA allowed us to reduce the number of variables and to create factors by grouping the highly related activities together. The PCA revealed a four-factor solution based on eigenvalues greater than 1. The results accounted for 56% of the variability. **Table 2** displays means and standard deviations of the items and their distribution into four factors. This four-factor solution highly resembles to LeFevre et al. (2009), which also accounted for 59% of the variability. Items that loaded on two factors were assigned to the factor on which the highest loading was observed, only if the difference in loadings on the other factor was more than 0.1. All items loaded 0.55 or higher on a factor, indicating a good description of the data. Most importantly, the PCA revealed similar factors as in LeFevre et al. (2009) and consequently the same labels were used: (1) number practices, (2) games, (3) number books, and (4) applications. The only difference was that two items (‘being timed’ and ‘making collections’) loaded onto the *games* factor in LeFevre et al.’s (2009) study, whereas they had higher loadings onto the *application* factor in our analysis. However, both factors fall into the ‘informal home numeracy activities’ as defined by LeFevre et al. (2009). For each factor, the means of the items belonging to that factor were computed and used in further analyses.

**Table 2:** Factor loadings and mean reported frequencies of home numeracy activities.

Items	Number practices	Number books	Games	Applications	*M*	*SD*
Identifying names of written numerals	0.81				3.01	1.18
Counting objects	0.73			0.35	3.60	1.17
Sorting things by size, color or shape	0.58			0.34	2.43	1.13
Learning simple sums	0.69				2.63	1.2
Writing numbers	0.63	0.49			2.43	1.2
Using number flashcards		0.60			1.66	0.93
Doing ‘connect the dot’ activities		0.71			1.76	0.83
Using number activity books		0.65			2.14	0.97
Reading number story books		0.70			1.74	0.95
Playing card games			0.87		2.33	1.06
Playing board games with dice or spinner			0.75		2.47	0.97
Talking about money when shopping				0.61	2.36	1.03
Measuring ingredient while cooking				0.60	1.93	0.98
Being timed				0.71	3.67	1.32
Collecting objects	0.34			0.63	2.63	1.31
Using calendars and dates				0.55	2.95	1.5

*Factor loadings < 0.3 are not displayed.*

### Basic Number Processing and Calculation Skills

In both comparison tasks, we first checked whether the magnitude representation was accessed by testing the ratio (non-symbolic comparison) and distance (symbolic comparison) effects (Moyer and Landauer, 1967; Halberda et al., 2008; Holloway and Ansari, 2009). Therefore, two repeated measures ANOVAs were conducted. A ratio effect was present in the non-symbolic comparison task, *F*(2,254) = 9.782, *p* < 0.001, $\eta_{p}^{2}$ = 0.120, indicating that children performed less accurate when the ratio between two numbers approached 1. A distance effect was found in the symbolic comparison task, *F*(1,127) = 36.499, *p* < 0.001, $\eta_{p}^{2}$ = 0.223, indicating that children performed more accurate when the distance between the two numbers was larger.

Although the program that kindergartens follow in Flanders was comparable for most schools, there might have been classroom differences that could affect children’s performance. Because children were recruited from only 10 classrooms, a one-way ANOVA was conducted on children’s basic number processing and calculation skills with classroom as between-subjects factor. This allowed us to examine whether the observed findings were affected by differences between classrooms. Results showed that on only one out of seven outcome measures (i.e., the connecting task; *p* = 0.035), children’s performance significantly differed between classrooms. No other statistical differences were observed (*p*s > 0.094). Therefore, classroom was not considered in the further analyses.

### Correlations

Partial correlations were computed controlling for sex, age, and maternal education, to examine the relationship between the home numeracy activities, children’s basic number processing skills and pictorial calculation skills (**Table 3**). It should be reminded that negative correlations were expected with the number line estimation tasks because they were indexed with percentage absolute error. Most importantly, the number practices factor was significantly correlated with the children’s performance in enumeration and symbolic number line estimation. These results indicate that the children who carried out more home numeracy activities with their parents, such as counting objects or learning simple sums, showed better performance in enumeration and symbolic number line estimation tasks. The number practices factor was not related to symbolic or non-symbolic comparison, non-symbolic number line estimation, and pictorial calculation skills in children. The games factor was significantly correlated with pictorial calculations only, whereas the applications factor (i.e., using numbers in daily life situations) was significantly correlated with symbolic number line estimation.

**Table 3:** Partial correlation coefficients (*p*-values) between the home numeracy activities, children’s basic number processing and calculation skills, controlled for sex, age, and maternal education.

Variables	1	2	3	4	5	6	7	8	9	10
**Home numeracy**										
1. Number practices										
2. Number books	0.49^∗∗^ (*p* < 0.001)									
3. Games	0.15 (0.113)	0.35^∗∗^ (*p* < 0.001)								
4. Applications	0.37^∗∗^ (*p* < 0.001)	0.28^∗∗^ (0.002)	0.25^∗∗^ (0.005)							
**Basic number processing**										
5. Sym NLE	–0.22^∗^ (0.016)	–0.16 (0.091)	–0.06 (0.538)	–0.24^∗∗^ (0.008)						
6. Non-sym NLE	–0.15 (0.111)	–0.02 (0.856)	0.03 (0.773)	–0.16 (0.077)	0.58^∗∗^ (*p* < 0.001)					
7. Sym comp.	0.02 (0.857)	–0.06 (0.531)	0.05 (0.591)	0.03 (0.739)	–0.16 (0.077)	–0.06 (0.511)				
8. Non-sym comp.	0.05 (0.588)	–0.03 (0.752)	–0.04 (0.666)	0.06 (0.487)	–0.02 (0.803)	–0.06 (0.547)	0.23^∗^ (0.013)			
9. Enumeration	0.21^∗^ (0.022)	0.12 (0.200)	0.14 (0.120)	0.06 (0.502)	–0.15 (0.102)	–0.13 (0.144)	0.23^∗^ (0.013)	0.20^∗^ (0.026)		
10. Connecting	0.15 (0.094)	0.15 (0.099)	0.11 (0.214)	0.13 (0.150)	–0.05 (0.566)	–0.06 (0.495)	0.19^∗^ (0.038)	–0.04 (0.662)	0.39^∗∗^ (*p* < 0.001)	
11. Pictorial cal.	–0.04 (0.652)	–0.00 (0.990)	0.18^∗^ (0.047)	–0.01 (0.939)	–0.11 (0.224)	–0.00 (0.996)	0.35^∗∗^ (*p* < 0.001)	0.06 (0.483)	0.22^∗^ (0.015)	0.31^∗∗^ (0.001)

*^∗^*p* < 0.05, ^∗∗^*p* < 0.01.*

*Sym comp., symbolic comparison; Non-sym comp., non-symbolic comparison; Sym NLE, symbolic number line estimation; Non-sym NLE, non-symbolic number line estimation; Pictorial cal., pictorial calculations subtest.*

Turning to the associations between the basic number processing skills in children (**Table 3**), we observed that enumeration was correlated with all the other tasks except with the symbolic and non-symbolic number line estimation. The connecting was correlated with enumeration and symbolic comparison. The symbolic comparison was correlated with all the other tasks except with the symbolic and non-symbolic number line estimation. The performance on the symbolic number line estimation correlated with non-symbolic number line estimation. Finally, children’s pictorial calculation skills were correlated with enumeration, connecting and symbolic comparison, but not with non-symbolic comparison and symbolic and non-symbolic number line estimation. It should be mentioned that the *p*-values were not adjusted for multiple comparisons, for which reason the current findings should be interpreted with caution.

Because we did not observe a relation between home numeracy, basic number processing and calculation skills, we did not investigate further whether there were any possible mediating effects of symbolic number processing and mapping skills on the relation between home numeracy and children’s calculation skills.

### Regressions

Three hierarchical regression analyses were conducted to further examine the unique contributions of the control variables and home numeracy factors on symbolic basic number processing and calculation skills (**Table 4**). The dependent and independent variables were determined based on the significant relationships observed in the correlation analysis. In the first regression, we examined the unique variance in symbolic number line estimation explained by ‘number practices’ and ‘applications’ after entering the control variables, sex, age, and maternal education. The control variables did not significantly contribute to the variance in symbolic number line estimation in the first step. However, the applications factor, but not number practices, accounted for 5.6% of the total variance in symbolic number line estimation in the final model. The second regression explored the unique variance in enumeration explained by ‘number practices.’ Control variables did not have an effect on enumeration. Number practices explained 6.4% of the total variance in enumeration. The last regression examined the unique variance in pictorial calculations explained by ‘games.’ The children’s age and maternal education were unique contributors to pictorial calculation, explaining 12.6% of the variance. Adding number practices as an additional factor increased the explained total variance to 15.5%.

**Table 4:** Hierarchical regression analyses examining the unique variance explained by the control variables and home numeracy factors in basic number processing and calculation skills.

Dependent variables	Steps	Independent variables	*B*	*SE (B)*	*β*	*p*	*Unique R^2^*
Symbolic NLE	1	Sex	–0.029	0.018	–0.14	0.11	0.019
		Age	–0.005	0.003	–0.18^∗^	0.04	0.033
		Maternal education	–0.000	0.011	0.00	0.98	0.039
	*F*_change_ (3,118) = 1.797, *p* = 0.15						0.044
	2	Number practices	–0.018	0.011	–0.15	0.11	0.047
		Applications	–0.024	0.012	–0.18^∗^	0.05	0.056
	*F*_change_ (2,116) = 4.978, *p* = 0.008						0.119
Enumeration	1	Sex	0.048	0.065	0.07	0.46	0.005
		Age	0.006	0.009	0.06	0.51	0.004
		Maternal education	0.053	0.040	0.12	0.19	0.021
	*F*_change_ < 1						0.020
	2	Number practices	0.089	0.038	0.21^∗^	0.02	0.043
	*F*_change_ (1,117) = 5.387, *p* = 0.02						0.064
Pictorial cal.	1	Sex	0.157	0.299	0.04	0.60	0.002
		Age	0.138	0.042	0.28^∗∗^	<0.001	0.078
		Maternal education	0.486	0.184	0.22^∗∗^	0.01	0.058
	*F*_change_ (3,118) = 5.664, *p* < 0.001						0.126
	2	Games	0.336	0.168	0.17^∗^	0.05	0.029
	*F*_change_ (1,117) = 4.015, *p* = 0.05						0.155

*^∗^*p* < 0.05, ^∗∗^*p* < 0.01.*

*Standardized betas from the last step in the regressions are reported.*

## Discussion

In the present study, we investigated whether the frequency of formal and informal numeracy activities at home was associated with children’s non-symbolic and symbolic number processing, mapping and calculation. We expected: (1) a relationship between home numeracy and symbolic number processing and mapping skills, but not with non-symbolic number processing skills; (2) an association between home numeracy and children’s calculation skills, and (3) a relationship between children’s calculation skills and symbolic number processing and mapping skills.

Correlation and regression analyses showed that, in line with the first hypothesized relation, home numeracy, in particular the number practices factor, was significantly associated with the children’s performance in enumeration (one of the mapping skills) but not with any other skills. Although, symbolic number line estimation (one of the symbolic number processing skills) was also significantly correlated with number practices, a regression analysis revealed that number practices did not explain unique variance in symbolic number line estimation performance. The associations between formal home numeracy and symbolic comparison and connecting were not significant. However, the correlation between ‘number practices’ and connecting showed a trend toward significance (**Table 3**). With a larger sample size, it is therefore plausible that also this correlation between ‘number practices’ and connecting would have been significant. Not only formal but also informal home numeracy, more specifically the applications factor, was significantly associated with symbolic number line estimation but not with any of the other tasks. Moreover, regression analysis showed that the number applications, together with children’s age, explained a unique variance in number line estimation over and above the number practices factor. Altogether, we observed that the symbolic comparison task was not related to either ‘number practices’ or ‘applications,’ although the symbolic number line estimation was. These results are in line with some previous findings. For example, a recent intervention study by Maertens et al. (2016) showed that children’s post-test scores on a comparison task did not significantly differ from pre-test after training, whereas their number line estimation scores improved. This idea is also theoretically supported by the finding that performances on comparison and number line estimation tasks are not associated with each other (Sasanguie and Reynvoet, 2013; but see Laski and Siegler, 2007). One possible explanation is that both tasks rely on different underlying mechanisms (Sasanguie and Reynvoet, 2013). Moreover, these findings are in line with Benavides-Varela et al. (2016) in the sense that home numeracy is not equally related to all basic number processing and calculation skills. They found that home numeracy was associated with children’s exact number skills but not with their approximate number processing. However, their measure of home numeracy, i.e., children’s self-reports about their knowledge of number related information such as phone numbers, birth dates, and number of siblings, was different than the commonly used questionnaires. Therefore these results are difficult to compare with other home numeracy research that has used questionnaires.

Children’s calculation (i.e., pictorial) was weakly but significantly related with informal home numeracy (i.e., games factor), together with children’s age and maternal education level. This finding is consistent with the idea that involvement of children in informal home numeracy activities, such as playing board or card games is beneficial for children’s acquisition of mathematical abilities (e.g., LeFevre et al., 2009; Niklas and Schneider, 2014). For example, Ramani and Siegler (2011) demonstrated that children who played a linear numerical board game improved more in mathematical skills over the course of 3 weeks compared to others who practiced other numerical activities. In the current study, calculation skills were measured with a subtest of the TediMath (i.e., pictorially presented addition and subtraction questions). The absence of the relation between formal home numeracy (i.e., number practices) and pictorial calculation skills can be explained by the parents’ selective attention for those home numeracy activities listed in the questionnaire that are more related to basic number processing skills than to calculation. This might be related to the age of the children in this study. For example, we observed that in the ‘number practices’ factor, the item ‘learning simple sums’ was reported significantly less frequently than the other activities such as ‘counting objects,’ *t*(126) = -8.10, *p* < 0.01 and ‘identifying names of written numerals,’ *t*(126) = -3.64, *p* < 0.01 (**Table 2**). We speculate that formal home numeracy activities, measured in a sample of children of about 5–6 years old, are related with children’s basic number processing skills, but not so much their (more advanced) pictorial calculation skills. Indeed, Ramani et al. (2015) demonstrated that formal home numeracy activities predicted basic number skills but not advanced skills in 3- to 5-year-old children (see also Manolitsis et al., 2013). Furthermore, the association between calculation (i.e., pictorial) skills and maternal education is also consistent with the earlier findings that maternal education influences children’s academic achievement (Davis-Kean, 2005).

Turning to our third hypothesis, the children’s pictorial calculation performance was associated with both mapping tasks and symbolic comparison task, but not with symbolic number line estimation or non-symbolic comparison and number line estimation. These findings are in line with previous studies (e.g., Holloway and Ansari, 2009; Mundy and Gilmore, 2009; Sasanguie et al., 2012a, 2013; Brankaer et al., 2014; Lyons et al., 2014; Vanbinst et al., 2015b; Mutaf Yıldız et al., 2018; for a meta-analysis, see Schneider et al., 2017) indicating that in particular symbolic skills are (predictively) related to mathematics achievement. Furthermore, the absence of the relation between pictorial calculations and symbolic number line estimation can be explained by the findings of Sasanguie et al. (2013). They showed that symbolic number line estimation was only related to a broad curriculum-based math test but not to a simple timed arithmetic test, although symbolic comparison was related to both types of mathematical measures (but see Booth and Siegler, 2008). Importantly, the PAE in the current study was comparable with previous studies investigating kindergartners’ number line estimation. Together, the findings suggest that the mathematical tasks used in the present study were age-appropriate. We did not observe any sequential relations between home numeracy, basic number processing, and pictorial calculation skills in children. Therefore, it was not useful to test whether symbolic number processing and mapping skills mediated the relation between home numeracy and children’s calculation skills.

### Limitations and Future Directions

This study holds some limitations. First, only one age group (i.e., last year kindergartners) was examined. It remains possible that different results emerge when examining the effect of home numeracy in younger or older children. For instance, in a study by Manolitsis et al. (2013), home numeracy measured at the start of kindergarten was not related to children’s mathematical skills at the start of kindergarten, although it predicted children’s math fluency at the end of first grade. Not only the relation between home numeracy and children’s mathematical skills might change over time, but also the frequency of the home numeracy activities. For instance, parents reported some activities, such as counting or reading number story books less frequently as their children became older (LeFevre et al., 2009; Hart et al., 2016). Second, the current sample consisted mainly of families with a middle-to-high SES. Several studies have already shown that SES affects children’s mathematical skills (Starkey et al., 2004; Krajewski and Schneider, 2009). Furthermore, the quality and quantity of mathematical support provided by the parents to their children is influenced by SES level (Starkey et al., 1999). Therefore, it remains an open question whether the current results can be generalized to low SES families. Third, another limitation of the current study is that the children’s general cognitive abilities (i.e., intelligence) were not assessed. Niklas and Schneider (2014) for instance observed that intelligence was an important predictor for mathematical skills, next to the home numeracy environment. However, other studies did not confirm this finding (e.g., Kleemans et al., 2012). Fourth, our study is correlational. It is therefore not possible to make causal inferences concerning the relation between home numeracy activities and basic number processing and calculation skills. To make causal claims, intervention studies are needed. In intervention studies, parents are informed about the role they play in the development of their children’s mathematical skills and how they can improve their support. Interestingly, such previous studies (Starkey and Klein, 2000; Sheldon and Epstein, 2005; Niklas et al., 2016) suggest that those interventions have a positive effect on mathematical skills.

Finally, it is important to consider that the questionnaire about informal home numeracy activities provides us with data on the frequency of how much a certain activity such as, ‘playing board games’ occurs. It does, however, not reveal information about the actual presence of numeracy talk in those activities. In fact, it is necessary to know the content and the amount of numeracy talk embedded in these home numeracy activities addressed in the questionnaire to profoundly interpret the results. A recent study showed that parents’ reports of home numeracy activities on a questionnaire and the amount of observed home numeracy talk during Lego building and book reading were not related (Mutaf Yıldız et al., 2018). Moreover, parents’ self-reports of home numeracy were positively correlated with children’s calculation skills whereas parents’ numeracy talk during Lego play correlated negatively with children’s calculation scores. We suggest that future studies should include both observations and questionnaires to better understand the content of the numeracy instructions in the home numeracy activities.

## Conclusion

Although the effects were small, the current findings are in line with the assumption that parents play a role in their children’s acquisition of basic number processing skills (LeFevre et al., 2009; Kleemans et al., 2012). More specifically, parents’ activities to practice numerical skills with their children, such as counting objects or writing numbers, are associated with their children’s symbolic number line estimation and enumeration skills. Overall, the present research demonstrated that disentangling children’s basic number processing skills and their calculation skills can be informative and might explain earlier reported contradictory findings on the association between home numeracy and mathematical abilities.

## Ethics Statement

The protocol was approved by the Social and Societal Ethics Committee of the KU Leuven. Consent forms were sent to the parents. If the parents agreed to participate, they were sent the questionnaires to fill in and their children were examined at their respective schools.

## Author Contributions

BMY, DS, BDS, and BR conceived and designed the study. BMY organized the data and ran the analyses. BMY, DS, BDS, and BR interpreted the results. BMY wrote the draft of the overall study. DS, BDS, and BR critically reviewed the draft. BMY revised the draft carefully.

## Conflict of Interest Statement

The authors declare that the research was conducted in the absence of any commercial or financial relationships that could be construed as a potential conflict of interest.

## Footnotes

1 On the enumeration task, 25% of the children (*n* = 32) scored 0. We computed correlation analyses with and without the 32 children. The significant relations between the enumeration task and the other variables did not change, thus we kept all the children in the subsequent analyses.
